# Preliminary study on the influencing factors of shear wave elastography for peripheral nerves in healthy population

**DOI:** 10.1038/s41598-021-84900-8

**Published:** 2021-03-10

**Authors:** Xinyi Tang, Bihui Zhu, Mei Tian, Ruiqian Guo, Songya Huang, Yuanjiao Tang, Li Qiu

**Affiliations:** Department of Medical Ultrasound, West China Hospital of Sichuan University, No.37 Guoxue Alley, Chengdu, 610041 Sichuan Province People’s Republic of China

**Keywords:** Peripheral nervous system, Stress and resilience, Ultrasound

## Abstract

This study took shear wave elastography (SWE) technology to measure the shear wave velocity (SWV) of peripheral nerve in healthy population, which represents the stiffness of the peripheral nerves, and research whether these parameters (location, age, sex, body mass index (BMI), the thickness and cross-sectional area(CSA) of the nerve) would affect the stiffness of the peripheral nerves. 105 healthy volunteers were enrolled in this study. We recorded the genders and ages of these volunteers, measured height and weight, calculated BMI, measured nerve thickness and CSA using high-frequency ultrasound (HFUS), and then, we measured and compared the SWV of the right median nerve at the middle of the forearm and at the proximal entrance of the carpal tunnel. The SWV of the median nerve of the left side was measured to explore whether there exist differences of SWV in bilateral median nerve. Additionally, we also measured the SWV of the right tibial nerve at the ankle canal to test whether there is any difference in shear wave velocity between different peripheral nerves. This study found that there existed significant differences of SWV between different sites in one nerve and between different peripheral nerves. No significant difference was found in SWV between bilateral median nerves. Additionally, the SWV of peripheral nerves was associated with gender, while not associated with age or BMI. The mean SWV of the studied male volunteers in median nerve were significantly higher than those of female (*p* < 0.05). Peripheral nerve SWE measurement in healthy people is affected by different sites, different nerves and genders, and not associated with age, BMI, nerve thickness or CSA.

## Introduction

Peripheral nerves are composed of nerve fibers, which transmit signals to muscles and control the autonomic nerve functions of the body. The evaluation of peripheral nerve function is of great importance for the diagnosis of neuromuscular diseases and the efficacy evaluation. Infections and gangrene are the main risk factors for lower extremity ulcers, and early detection and treatment of diabetic peripheral neuropathy (DPN) can significantly reduce the occurrence of lower extremity ulcers and amputations, thus improving the patients’ quality of life. At present, there is no gold standard for the diagnosis of peripheral nerve diseases, which mainly depends on clinical symptoms, signs, neurophysiological examinations, quantitative sensory examinations, and magnetic resonance imaging (MRI). A neurophysiological examination can provide data on the functional status of neurons, peripheral nerves, neuromuscular junctions and muscles^1^; however, this method is invasive and time-consuming. Quantitative sensory examination is used to evaluate the degree of peripheral neurosensory disorder, but it is susceptible to a variety of subjective and objective factors such as analysis software, subject cooperation, and working experience of the testers. MRI can describe the morphological characteristics of peripheral nerves, but it is susceptible to physical factors such as bones or internal fixation, higher price and artifacts. Traditional high-frequency ultrasound (HFUS) can reveal the morphological changes of peripheral nerves, and detect the continuity, thickness, echo intensity, and cross-sectional area (CSA) of peripheral nerves^2^. It is convenient and non-invasive; nonetheless, the results can only reflect the morphological characteristics of peripheral nerves.

Ultrasound elastography is a technology based on biomechanical properties where the structure and composition of tissue determine its deformation and rebound capabilities. This elastic feature can be used to evaluate the target tissue when it is subjected to external or internal stimuli, and the consequent value can reflect the tissue stiffness^3^. At present, the main elastography technologies include strain elastography (SE)^4^, shear wave elastography (SWE), and acoustic radiation force pulse imaging (ARFI)^5^. SWE is a new technique for evaluation of tissue stiffness, which can quantitatively measure the stiffness of the object. It has been widely applied in organs like liver, kidney, breast, or thyroid, and in the musculoskeletal system, including muscles and tendons^4, 6–9^. Currently, researchers are exploring new applications for elastography, such as diagnosis of peripheral neuropathy including DPN and carpal tunnel syndrome. Previous research reveals that diseases such as carpal tunnel syndrome and DPN can cause structural changes, edema within the nerve fascicle and reduction of blood flow, leading to the compression and increase in stiffness of the affected nerve^10, 11^, which provide the possibility of diagnosis with SWE. At the same time, some studies have preliminarily confirmed that SWE can objectively reflect the increase of in stiffness of nerve tissue in indentation testing^12^. Yet, for such a new technology, there are relatively few studies on shear wave elastography of peripheral nerves, especially those addressing the methodology and influencing factors of peripheral nerve SWE in healthy people.

The propagation velocity of the shear wave in tissue can be accurately quantified, and then calculated as follows: E = 3ρC^2^, where E is named Young's modulus, representing the mechanical properties of the tissue, ρ denotes the density of tissue, and C is the shear wave velocity (SWV)^13^. The harder the tissue, the faster the SWV, the larger the Young's modulus value. However, the premise of applying the above-mentioned formula is typically in isotropic, elastic and locally homogeneous tissues, such as the liver, thyroid, or breast. Muscles, tendons, and peripheral nerves are obviously anisotropic tissues. Therefore, substantial studies have pointed out that when using SWE to measure these anisotropic tissues, the SWV is more valuable than the Young's modulus value^14, 15^. In addition, gender, age, height, weight, and BMI were included in previous studies on SWE of musculoskeletal system. Bortolotto et al.^16^ found the stiffness of the median nerve differed among different portions. Yang et al.^17^ found that there were differences in skin stiffness between different genders. In the research of Bedewi et al.^18^, significant inverse correlation with height and weight was noted at the C6 nerve root. Although there were studies simply discussing some issues, the influence factors on the elasticity of peripheral nerve have been seldom investigated systematically. Therefore, we intended to apply SWE technology to explore whether parameters such as location, age, sex, body mass index (BMI), the thickness and CSA of the peripheral nerve can affect the stiffness of the median nerve and the tibial nerve through measuring the SWV in healthy population, which may provide the primary reference and objective evidence for SWE measurement in peripheral nerve disorders.

## Materials and methods

### Patient population

A total of 105 healthy volunteers, who were recruited at our institution between September 2018 and December 2018, were enrolled in this cross-sectional study. The gender, age, height, weight, and BMI were recorded, nerve thickness and CSA were measured using HFUS, and SWV was measured in SWE mode.

This study was approved by the Ethics Committee of West China Hospital of Sichuan University and performed in accordance with the Declaration of Helsinki, 2013. Primary inclusion criteria for the healthy volunteers were: (1) age > 18 years old, (2) cooperating with the US examination; while the exclusion criteria were: (1) pregnancy, (2) suffering from diseases such as systemic neurological diseases, post-traumatic changes, nerve entrapment syndrome, musculoskeletal diseases or systemic metabolic diseases, which may cause peripheral nerve abnormalities, (3) local inflammation, trauma, scar, tumor or history of surgery in check regions. Volunteers were classified into three groups according to age: 18 ~ 39 years, 40 ~ 59 years and over 60 years, and were divided into three groups according to BMI: BMI < 18.5 kg/m^2^, 18.5 ≤ BMI < 24 kg/m^2^, and BMI ≥ 24 kg/m^2^. All of these volunteers were randomly assigned to two experienced doctors at the Department of Ultrasound at our institution. Our previous study, which had a small sample size, initially confirmed excellent inter- and intra-observer agreements^19^, thus, the feasibility and reliability were not discussed in this article.

According to the previous article, the Pearson correlation coefficient of the main relevant factors and the SWV for the median nerve in healthy people floated between 0.45 and 0.6^20^. We assumed that power reached 95% and α = 0.025. In the most conservative case, we set R = 0.45 in the calculation. The sample size, which was calculated using the PASS15 software, was set to 67. Even with a 20% dropout rate, 84 volunteers were sufficient. In order to ensure that each subgroup could have sufficient sample for statistical analyses, a total of 110 volunteers were recruited, among whom 5 were excluded due to missing data (4 for missing data on nerve thickness, 1 for missing data on height and weight).

### High-frequency US and SWE examination

**Figure 1:**
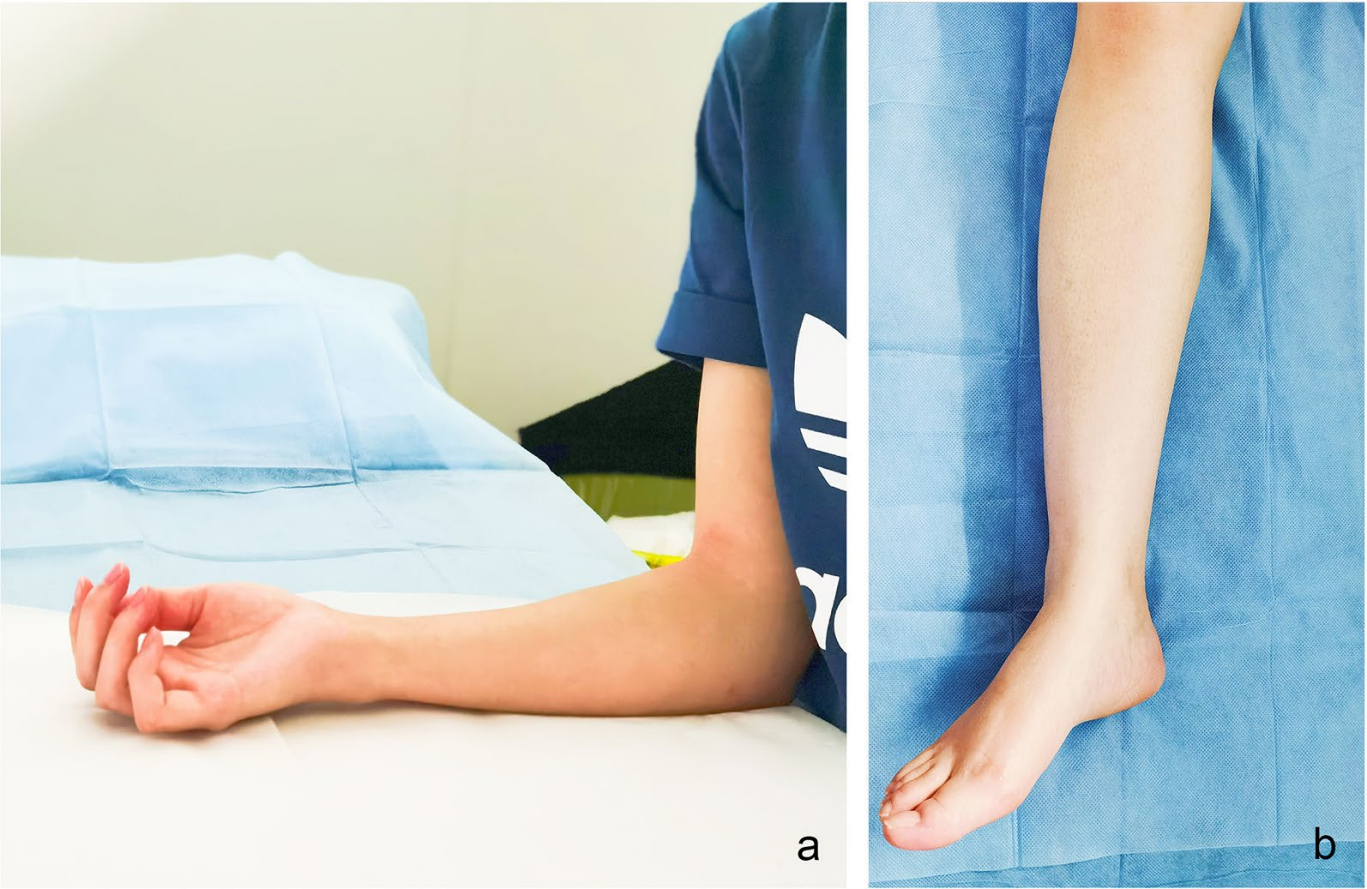
Examples of the position during the assessment. (**a**) the subjects were asked to sit facing the examiner in a neutral position and to keep their forearm in the supine position, upper arms close to the chest wall, the elbow flexed 90°, upper limbs relaxed and fingers naturally semi-flexed during the median nerve scanning; (**b**) the subjects were asked to lie in the supine position. The ankles were positioned in slight plantar flexion and were slightly rotated externally and the lower extremity was in a neutral position during the tibial nerve scanning.

During examinations, the room was temperature-controlled (21–25 °C) and silence maintained. To obtain images of the median nerve, each subject was asked to sit facing the examiner in a neutral position and to keep their forearm in the supine position, upper arms close to the chest wall, the elbow flexed 90°, upper limbs relaxed and fingers naturally semi-flexed. The examined limb was carefully maintained in a neutral position without any movement^19^. For the tibial nerve scanning, the subjects were asked to lie in the supine position. The ankles were positioned in slight plantar flexion and were slightly rotated externally and the lower extremity was in a neutral position^10^. (As is shown in Fig. 1).

In this study, bilateral median nerves and right tibial nerve were measured. For the median nerve, two sites were scanned: the middle of the forearm and the proximal entrance of the carpal tunnel. We made marks at the midpoint of the line between scaphoid bone and pisiform bone and the midpoint of the line from radial head to the styloid process of the radius, and then at these two sites, we first located the median nerve at the proximal entrance of the carpal tunnel and at forearm on the transverse imaging plane, respectively. After locating the nerve, we measured the nerve thickness and CSA in short-axis. Finally, the probe was rotated 90° to obtain the longitudinal image, where we measure the SWV of the nerve in the SWE mode. For the tibial nerve, we made a mark at 4 cm proximal to the medial malleolus^10^ to locate the tibial nerve at the tibial ankle canal, and the following steps were similar to those of the median nerve scanning.

The used sonographic equipment was Aixplorer US system (SuperSonic Imagine, Aixen-Provence, France. Version: 12.3.0.841 + SP1. Spatial resolution: < 1 mm; temporal resolution: > 8 Hz for frame rate), with an SL 15–4 multifrequency linear transducer, and the depth was fixed at 2–3 cm. During the entire US scanning, the tip of the probe was covered with several millimeters of bubble-free ultrasonic gel and was then perpendicularly and smoothly placed to the skin. The operator should avoid applying pressure on the skin. In the SWE mode, the superficial musculo-skeletal pre-set shall be selected in the standard default mode, and the maximal scale was adjusted to 600 kPa. The ROI was moved to include the scanned nerves and the circular Q-box was placed in the measured nerve and was fixed to a diameter of 2 or 1 mm according to the thickness of the nerves^21–23^, for the epineurium should not be included. The system automatically generates the SWV of the median and tibial nerves, including the maximum shear wave velocity (Cmax), average shear wave velocity (Cmean), and minimum shear wave velocity (Cmin). Each point was measured three times, and the average value was calculated and recorded. All of these velocity results were measured in m/s.

### Statistical analysis

Statistical analyses were performed in SPSS software (Version 24.0; IBM, NY). Variables are expressed as median (interquartile range). The differences in age, height and BMI in volunteers were evaluated using the Mann–Whitney U test. The SWV of the median nerve in the bilateral forearm, the SWV of the median nerve in different sites of right side, and the SWV of the right median nerve and the right tibial nerve were compared using the Wilcoxon signed-rank test. The differences in SWV, CSA and nerve thickness between different genders were compared using the Mann–Whitney U test, and the differences between different ages and BMI groups were compared using Kruskal–Wallis test. Additionally, the correlation analysis between SWV and nerve thickness and CSA was undertaken using the Spearman’s correlation test. *p *value of < 0.05 was considered as the statistically significant difference.

### Ethical statement

The authors are accountable for all aspects of the work in ensuring that questions related to the accuracy or integrity of any part of the work are appropriately investigated and resolved. The study was approved by the West China Hospital of Sichuan University Ethics Committee. Informed consent was obtained from all volunteers.

## Results

This study contained 105 healthy volunteers, 49 males, and 56 females. 5 out of 110 initially recruited volunteers were excluded due to missing data (one of whom withdrew halfway due to personal work arrangement, resulting in the absence of height and weight data, and four of whom lost their cross-sectional HFUS data due to technical malfunction). And the demographic data of the volunteers in this study are summarized in Table 1. The median age of the participants was 49.00 (27.50) years and the median BMI was 22.43 (3.59) kg/m^2^. The height (*p* < 0.001), weight (*p* < 0.001), and BMI (*p* = 0.033) of male volunteers were higher than those of female volunteers. Volunteers were divided into three groups based on age: 32 cases in the 18–39 years old group, 49 cases in the 40–59 years old group, and 24 cases in the ≥ 60 years old group. According to BMI, volunteers were divided into three groups: 6 cases in the BMI < 18.5 kg/m^2^ group, 68 cases in the 18.5 ≤ BMI < 24 kg/m^2^ group, and 31 cases in the BMI ≥ 24 kg/m^2^ group.

**Table 1:** The baseline demographic data of the volunteers in this study.

Characteristic	Value	*p*
Volunteers	105	
Male	49	
Female	56	
Hand dominance (left:right)	1:104	
**Age, y**		
All volunteers	49.00 (27.50)	0.163
Male	50.00 (24.50)	
Female	47.00 (23.75)	
**Height, m**		
All volunteers	1.60 (0.11)	< 0.001
Male	1.65 (0.12)	
Female	1.55 (0.08)	
**Weight, kg**		
All volunteers	59.00 (12.00)	< 0.001
Male	65.00 (11.25)	
Female	54.50 (9.00)	
**BMI, kg/m**^**2**^		
All volunteers	22.43 (3.59)	0.033
Male	23.44 (3.74)	
Female	22.23 (3.72)	

**Figure 2:**
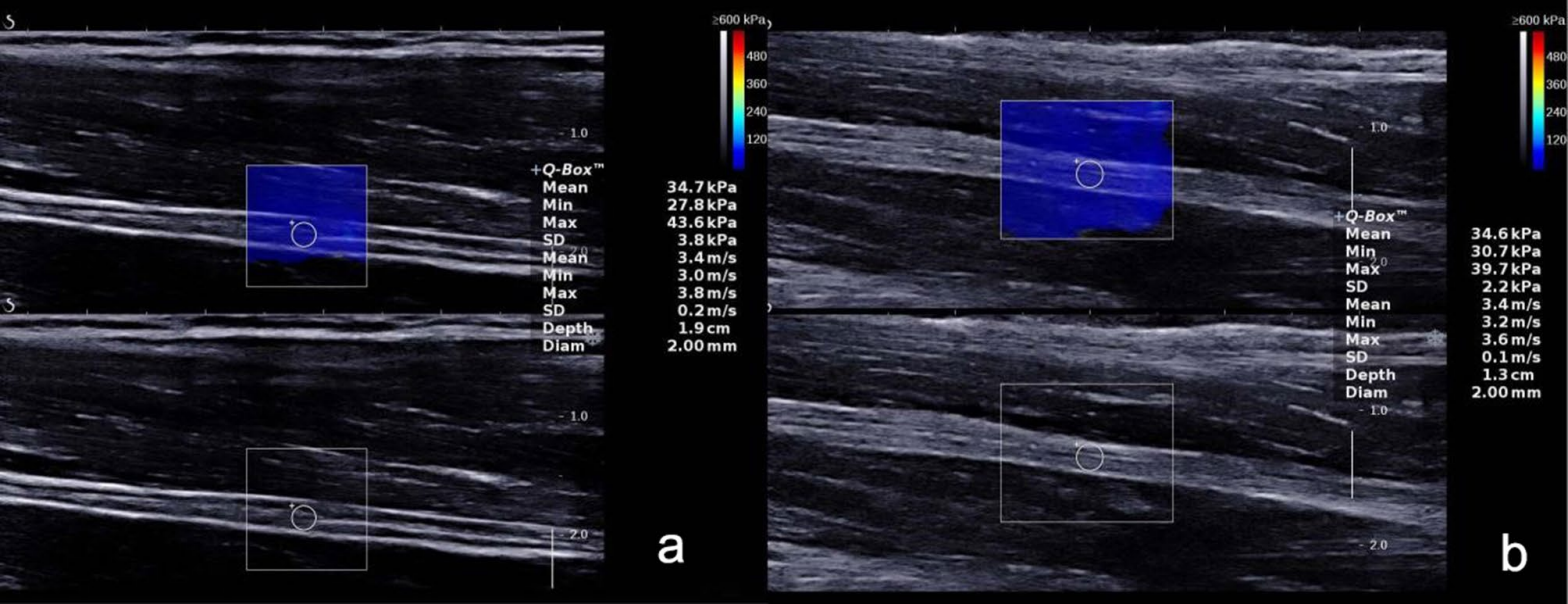
In the relaxed position, the shear wave velocity of the right (**a**) and left (**b**) median nerve forearm segment.

**Figure 3:**
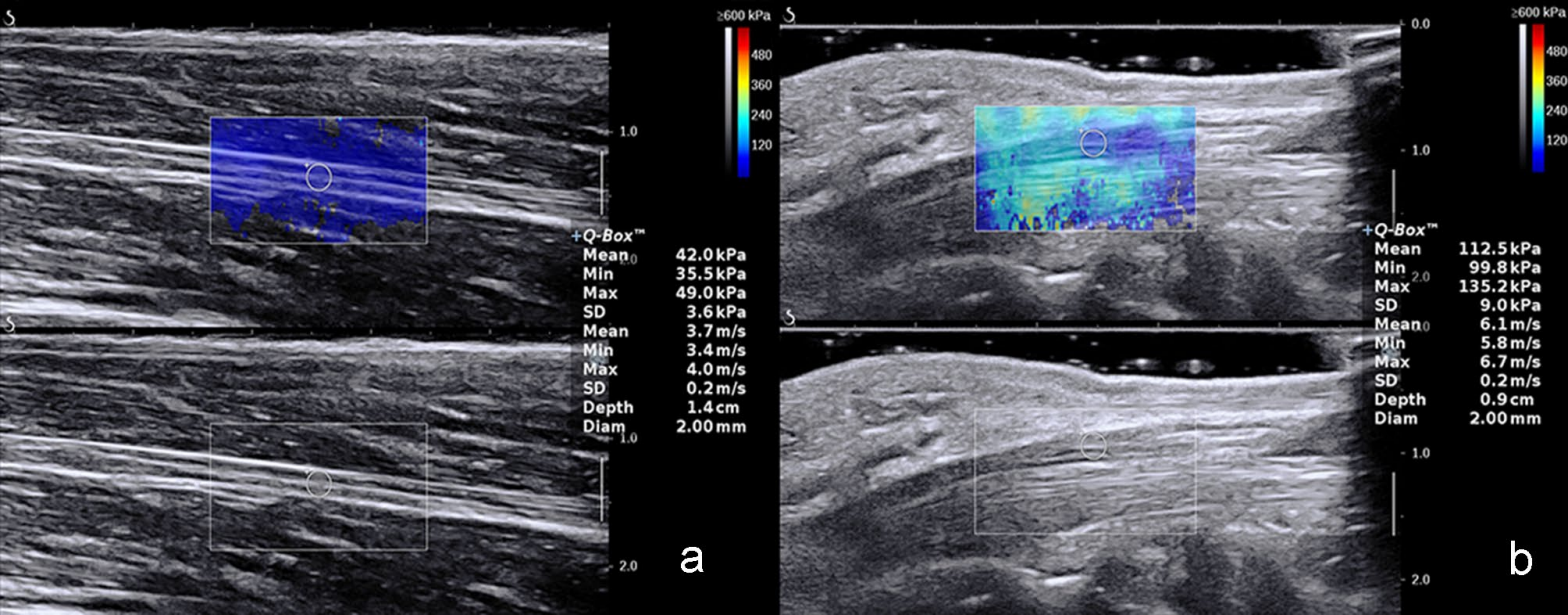
In the relaxed position, the shear wave velocity of the right median nerve at forearm (**a**) and at the carpal tunnel (**b**).

Figure 2 and Table 2 showed that there were no significant differences in the mean, minimal or maximal SWV between the right and left median nerve in the forearm. Figure 3 and Table 3 showed that the mean, minimal or maximal SWV at the carpal tunnel of the right median nerve were all higher compared to those of the forearm; the observed differences were statistically significant (*p* < 0.001).

**Table 2:** Comparison of bilateral median nerve shear wave velocity in the same volunteer.

Shear wave velocity	Right	Left	*p*
Cmean (m/s)	3.37 (0.76)	3.53 (0.52)	0.146
Cmin (m/s)	3.10 (0.75)	3.23 (0.60)	0.313
Cmax (m/s)	3.77 (0.83)	3.90 (0.63)	0.126

**Table 3:** Comparison of median nerve shear wave velocity in different parts.

Shear wave velocity	At forearm	At carpal tunnel	*p*
Cmean (m/s)	3.37 (0.76)	4.30 (0.80)	< 0.001
Cmin (m/s)	3.10 (0.75)	3.67 (0.87)	< 0.001
Cmax (m/s)	3.77 (0.83)	5.13 (1.00)	< 0.001

**Figure 4:**
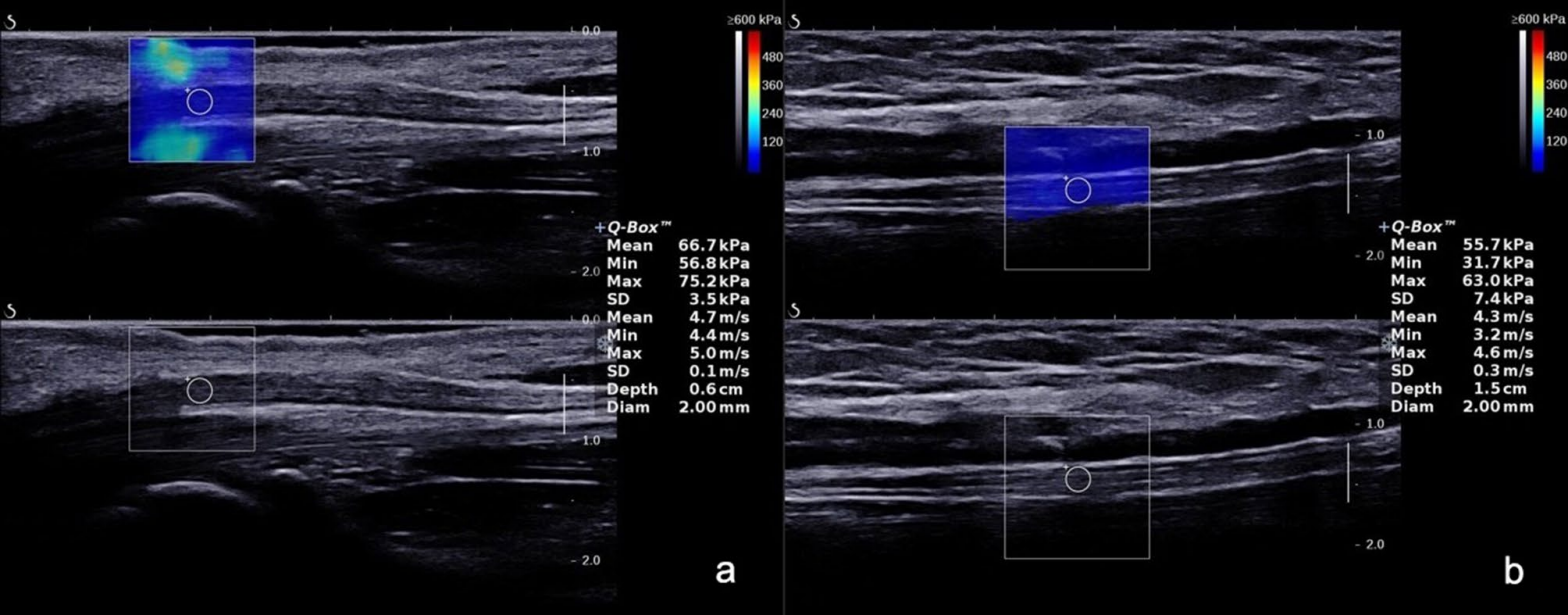
In the relaxed position, the shear wave velocity of the median nerve at the carpal tunnel (**a**) and the tibial nerve at the tarsal tunnel (**b**).

**Figure 5:**
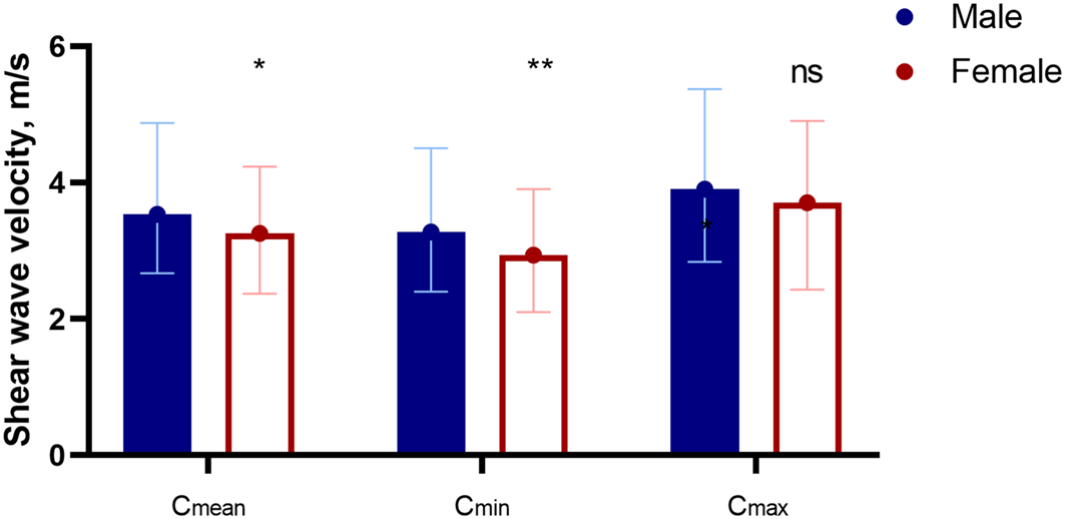
Comparison of shear wave velocities in relation to gender. *Means *p* < 0.05, and **means *p* < 0.01.ns means not significant.

Furthermore, the mean, minimal or maximal SWV of the right median nerve at the carpal tunnel were 4.30 (0.80) m/s, 3.67 (0.87) m/s, 5.13 (1.00) m/s, respectively and the mean, minimal or maximal SWV of the right tibial nerve at the tarsal tunnel were 3.87 (0.76) m/s, 3.37 (0.84) m/s, 4.30 (0.75) m/s, respectively (Fig. 4 and Table 4). The SWV of the right median nerve at the carpal tunnel were significantly higher compared to the right tibial nerve at the tarsal tunnel (*p* < 0.001). Besides, the mean and minimal SWV of the studied male volunteers in the right median nerve were significantly higher compared to female volunteers (*p* < 0.05), while no significance was noted in the maximal SWV . (Table 5 and Fig. 5).

**Table 4:** Comparison of shear wave velocities of different peripheral nerves.

Shear wave velocity	Median nerve (at the carpal tunnel)	Tibial nerve (at the tarsal tunnel)	*p*
Cmean (m/s)	4.30 (0.80)	3.87 (0.76)	< 0.001
Cmin (m/s)	3.67 (0.87)	3.37 (0.84)	< 0.001
Cmax (m/s)	5.13 (1.00)	4.30 (0.75)	< 0.001

**Table 5:** Comparison of shear wave velocities of right median nerve at forearm, nerve thickness and cross-sectional area in different genders.

Gender	N	Shear wave velocities			Nerve thickness (Right median nerve in forearm, mm)	Cross-sectional area (Right median nerve in forearm, mm^2^)
		Cmean (m/s)	Cmin (m/s)	Cmax (m/s)		
Male	49	3.53 (0.64)	3.27 (0.72)	3.90 (0.80)	2.40 (0.45)	10.20 (1.80)
Female	56	3.25 (0.66)	2.93 (0.74)	3.70 (0.81)	2.10 (0.40)	9.60 (1.68)
*p*		0.016	0.004	0.060	< 0.001	0.005

At the same time, we found a significant difference between two genders in nerve thickness and CSA (Table 5). However, the results of further correlation analysis suggested that the higher SWV were not associated with the higher nerve thickness and CSA (Table 6).

**Table 6:** Correlations between shear wave velocities and nerve thickness and cross-sectional area.

Shear wave velocity	Nerve thickness		Cross-sectional area	
	Spearman correlation coefficient	*p*	Spearman correlation coefficient	*p*
Cmean	0.111	0.259	− 0.100	0.308
Cmin	0.089	0.365	− 0.105	0.285
Cmax	0.102	0.301	− 0.057	0.563

Our results showed no statistically significant differences in the SWV of the right median nerves in the middle forearm between groups of different age and BMI (Table 7).

**Table 7:** Comparison of shear wave velocities of right median nerve at forearm in different age and BMI groups.

Groups		N	Mean shear wave velocity (Cmean) (m/s)	*p*
Age (years old)	18–39	32	3.37 (0.78)	0.857
	40–59	49	3.37 (0.70)	
	≥ 60	24	3.55 (0.84)	
BMI (kg/m^2^)	< 18.5	6	3.75 (1.21)	0.587
	18.5–24	68	3.35 (0.65)	
	≥ 24	31	3.53 (0.74)	

Based on our research results, we listed the median and percentiles values of SWV of median nerves in forearm and at the carpal tunnel and tibial nerve at the tarsal tunnel respectively for male and female (Table 8).

**Table 8:** The reference range of mean shear wave velocities of peripheral nerves of different sites.

Site	Gender	Mean shear wave velocity (Cmean) (m/s)	
		Median	Percentiles (25–75)
Right median nerve	Male	3.53	3.24–3.87
In forearm^a^	Female	3.25	3.01–3.67
Right median nerve	Male	4.23	3.93–4.59
At the carpal tunnel	Female	4.35	4.01–4.92
Right tibial nerve	Male	4.00	3.62–4.27
At the tarsal tunnel^a^	Female	3.72	3.28–4.06

^a^*p* < 0.01.

## Discussion

In tendon and muscle, shear waves propagate faster through stiffer contracted tissue, as well as along the long axis of tendon and muscle. For peripheral nerves, the outermost layer is the fibrous connective tissue referred to as the epineurium, and individual nerve fibers inside are organized into individual bundles, called fascicles^24^. Similar structures suggest that the mode of shear wave propagation in peripheral nerves is similar to that in muscles and tendon, that is, shear waves propagate along the long axis of peripheral nerves and increased as the nerves get stiffer^25^. Peripheral nerve disease is a structure and/or dysfunction disorder of peripheral sensory, motor, and autonomic nerves caused by a variety of causes. Patients with peripheral neuropathy suffer from nerves that are prone to edema, fibrosis, calcification, all of which can eventually cause changes in stiffness^26^. HFUS has been used for the diagnosis of peripheral neuropathy such as peripheral nerve injury, peripheral nerve entrapment, diabetic peripheral neuritis, and similar for quite a long time. However, HFUS can only reflect the changes in the morphology of peripheral nerves and the blood flow signal, while it cannot reflect the changes in tissue stiffness. Accurate quantification of the stiffness of the affected nerves is of considerable significance since it can be applied for disease diagnosis, efficacy evaluation and follow-up, and this is the reason why we chose shear wave elastography to evaluate peripheral nerve.

The present study found significant differences in the SWV between different sites in one nerve, as well as significant differences between different nerves. No significant difference was found in the SWV between bilateral median nerves. Additionally, the SWV was associated with gender, while it was not associated with age or BMI.

The differences in the mean, minimum, and maximum SWV of the bilateral median nerve were not statistically significant (*p* > 0.05), thus indicating that there was no significant difference in the stiffness of the bilateral median nerve in healthy people, which was consistent with results reported by Andreisek et al.^27^. This suggests that if there is a difference in SWV between bilateral symmetrical positions, it should result from the neuropathy, not the difference between the left and right sides^28^. Therefore, for patients with unilateral neuropathy, the contralateral SWV can be used as a reference. However, in some cases, such as DPN, which often has symmetrical onset, it is necessary to establish a reference range of normal SWV value to determine whether there is a change in nerve stiffness.

By comparing the right median nerve at the carpal tunnel with the segment at the middle of the forearm, we found a significant difference in SWV between the two sites (*p* < 0.001), with a higher SWV at the carpal tunnel. Tissue SWE measurement may be affected by surrounding tissues, especially harder tissues such as bones and tendons. Compared with the forearm, the structure of the carpal tunnel is complex, including the transverse carpal ligament and the flexor tendon. The SWE measurement of the nerve may be affected by the complex structure of the carpal tunnel, which may explain the higher SWE measurement results of the median nerve at the carpal tunnel. Besides, the bone may have a certain effect on the measurement of peripheral nerves^16^, and the nerve may be more affected by the bone structure in the wrist, thus resulting in a higher SWV at the carpal tunnel than at the forearm segment. At the same time, the depths of the median nerve at these two sites are not the same, though the probe pressure is very small and similar in every measurement, the median nerve at the superficial position may be subjected to greater probe pressure, which can partially explain the observed difference. This finding suggests that the SWE measurement of the same peripheral nerve may be different in different segments. In clinical application, when measuring peripheral nerve stiffness in different patients, the same segment should be selected to obtain comparable results.

This study compared the SWV at the right medial nerve carpal tunnel and the right tibial nerve ankle canal, and found that the maximum, minimum, and average SWV at the right median nerve carpal tunnel were greater than those at the right tibial nerve at the ankle canal; the observed differences were statistically significant (*p* < 0.001). The anatomic structure of the carpal tunnel is more complicated than that of the ankle canal. The transverse carpal ligament with a large number of tendons running through it maintains pressure at the carpal tunnel. This complex anatomic structure has an impact on the SWV, which may explain the observed difference. Therefore, corresponding normal ranges of SWV should be separately established to accurately evaluate peripheral neuropathy for different peripheral nerves.

In our study, we also found statistically significant differences (*p* < 0.05) of the mean SWV in the middle of the forearm of the right median nerves between males and females. SWV of the median nerve were greater in men than in women. This suggests that when setting the reference value range, it should be formulated separately according to gender. However, further analyses revealed that gender, nerve thickness, and CSA are significantly related. Men's peripheral nerves are larger than women's, but the correlations between nerve thickness and CSA and SWV were not significant, indicating that the differences in SWV in people of different genders were not caused by different nerve thickness or CSA. We assume this could be due to the thickness of adipose tissue that can affect the shear wave measurement^29^, and since women generally have thicker subcutaneous fat layers, they have lower nerve stiffness compared to men. Therefore, in the clinical use of elastography to evaluate peripheral nerves, the impact of gender on nerve stiffness measurements should be considered.

Previous studies have found that in some musculoskeletal tissues, such as tendon tissue, stiffness decreases with age^30^. Other studies have shown that the SWV of peripheral nerves in subjects over 40 years of age has a downward trend^20, 31^. This was not consistent with our results, since the differences in the maximum, minimum, and mean SWV of the right median nerve forearm were not statistically significant (*p* > 0.05) in different age groups. Bortolotto and others used SWE to study the stiffness of normal median nerves in adults, finding that the stiffness of median nerves was not related to the age of the subjects^16^, which is consistent with our results. Additionally, our study showed that there was no statistical significance (*p* > 0.05) in SWV of the median nerve between different BMI groups, which is consistent with the previous study on the adult median nerve and tibial nerve^20^. This suggests that the SWV of peripheral nerves is not significantly affected by age and BMI. Thus, it is not necessary to establish a reference value range for different age and BMI groups.

There are some limitations in our study: (1) even though our results confirm our conjecture to a certain extent, they cannot be used as a reference value range. For the purpose of establishing a range of normal reference values, the statistical population should contain the entire population, i.e. the sample size should be larger, and the sampling methods should also be stricter. (2) We did not compare different methods of measuring peripheral nerve stiffness. (3) The number of measured sites was limited, which should be addressed by further research. (4) We have found the effect of gender on the SWV of nerves, but our current research cannot elucidate it accurately. (5) None among 105 volunteers included in this study had BMI exceeding 30 kg/m^2^. The absence of obese people in this study makes it difficult to judge whether the extreme BMI condition would cause the differences in SWV.

## Conclusion

Peripheral nerve SWE measurement in healthy people was affected by different sites, different nerves, and different genders, while it was not associated with age, BMI, nerve thickness or CSA. The reference range of SWE for normal peripheral nerves should be established for the related parameters. When conducting an SWE study on peripheral neuropathy, an appropriate control group should be established based on the influencing factors.
